# Effects of a Low Dose of T-2 Toxin on the Percentage of T and B Lymphocytes and Cytokine Secretion in the Porcine Ileal Wall

**DOI:** 10.3390/toxins13040277

**Published:** 2021-04-13

**Authors:** Paweł Wojtacha, Wojciech Trybowski, Piotr Podlasz, Magdalena Żmigrodzka, Józef Tyburski, Magdalena Polak-Śliwińska, Ewa Jakimiuk, Tadeusz Bakuła, Mirosław Baranowski, Krystyna Żuk-Gołaszewska, Łukasz Zielonka, Kazimierz Obremski

**Affiliations:** 1 Department of Industrial and Food Microbiology, Faculty of Food Science, University of Warmia and Mazury in Olsztyn, 10-726 Olsztyn, Poland; pawel.wojtacha@uwm.edu.pl; 2 Regional Veterinary Inspectorate in Gdańsk, 80-958 Gdańsk, Poland; wtrybowski@gmail.com; 3 Department of Pathophysiology, Forensic Veterinary Medicine and Administration, Faculty of Veterinary Medicine, University of Warmia and Mazury in Olsztyn, 10-718 Olsztyn, Poland; 4 Department of Pathology and Veterinary Diagnostics, Institute of Veterinary Medicine, Warsaw University of Life Sciences—SGGW, 02-776 Warsaw, Poland; magdalena_zmigrodzka@sggw.edu.pl; 5 Department of Agroecosystems and Horticulture, Faculty of Agriculture and Forestry, University of Warmia and Mazury in Olsztyn, 10-719 Olsztyn, Poland; jozef.tyburski@uwm.edu.pl; 6 Department of Commodity Science and Food Analysis, Faculty of Food Science, University of Warmia and Mazury in Olsztyn, 10-726 Olsztyn, Poland; m.polak@uwm.edu.pl; 7 Department of Veterinary Prevention and Feed Hygiene, Faculty of Veterinary Medicine, University of Warmia and Mazury in Olsztyn, 10-718 Olsztyn, Poland; ewa.jakimiuk@uwm.edu.pl (E.J.); bakta@uwm.edu.pl (T.B.); kerim@uwm.edu.pl (M.B.); lukasz.zielonka@uwm.edu.pl (Ł.Z.); 8 Department of Agrotechnology and Agribusines, Faculty of Agriculture and Forestry, University of Warmia and Mazury in Olsztyn, 10-719 Olsztyn, Poland; kzg@uwm.edu.pl

**Keywords:** T-2 toxin, prepubertal gilts, GALT, lymphocytes, cytokine

## Abstract

Plant materials used in the production of pig feed are frequently contaminated with mycotoxins. T-2 toxin is a secondary metabolite of selected *Fusarium* species, and it can exert a harmful influence on living organisms. Most mycotoxins enter the body via the gastrointestinal tract, and they can modulate the gut-associated lymphoid tissue (GALT) function. However, little is known about the influence of low T-2 toxin doses on GALT. Therefore, the aim of this study was to evaluate the effect of T-2 toxin administered at 50% of the lowest-observed-adverse-effect level (LOAEL) on the percentage of CD2+ T cells, CD4+ T helper cells, CD8+ cytotoxic T cells, CD4+CD8+ double-positive T cells, TCRγδ+ cells, CD5+CD8- B1 cells, and CD21+ B2 cells, and the secretion of proinflammatory (IFN-γ, IL-1β, IL-2, IL-12/23p40, IL-17A), anti-inflammatory, and regulatory (IL-4, IL-10, TGF-β) cytokines in the porcine ileal wall. The results of the study revealed that T-2 toxin disrupts the development of tolerance to food antigens by enhancing the secretion of proinflammatory and regulatory cytokines and decreasing the production of anti-inflammatory TGF-β. T-2 toxin triggered the cellular response, which was manifested by an increase in the percentage of CD8+ T cells and a decrease in the percentage of B2 and Tγδ lymphocytes.

## 1. Introduction

Mycotoxins are secondary fungal metabolites that can exert multidirectional adverse effects on living organisms. These substances are derived from main metabolic pathways and play various roles in fungal biology. Secondary metabolism in molds is associated with fungal development. In many cases, the benefit these compounds confer on the organism is unknown [1]. Secondary metabolism is commonly associated with sporulation processes in fungi. Some mycotoxins might function as virulence factors, or their presence could give a competitive edge to the producing organism (defense and weapons against fungivorous predators) or enhance the survivability of spores or induce sporulation and enhance perithecial formation (e.g., zearalenone) [2]. Fungal secondary metabolites are harmful to humans, animals, plants, and microorganisms [3,4] because acute and chronic exposure to these substances can damage the liver and/or kidneys and impair the function of these organs [5,6].

T-2 toxin and its deacylated form, HT-2 toxin, occur in most climatic zones, and together with diacetoxyscirpenol and neosolaniol, they belong to the group of type A trichothecenes (TCHA) [7]. These mycotoxins are produced by fungi of the genera *Fusarium*, *Myrothecium*, *Spicellum*, *Stachybotrys*, *Cephalosporium*, and *Trichothecium* [8], as well as sac fungi of the phylum *Ascomycota* which infect field-grown corn, oats, barley, wheat, rye, sorghum, and rice [9]. T-2 toxin is accumulated mainly in cereal grain, but due to its resistance to high temperature, the toxin is not completely eliminated during high-temperature processing, and it can contaminate foodstuffs and feedstuffs [5,10].

Prolonged exposure to TCH can decrease appetite and lead to weight loss in humans and animals. Characteristic inflammatory changes, ulcers, and necrotic changes are observed in oral and esophageal mucosa of infected individuals. T-2 toxin exerts numerous adverse effects, including anorexia, vomiting, retarded growth, immunosuppression, and neuroendocrine changes [11,12]. It has been identified as an etiological factor in gastrointestinal disorders such as alimentary toxic aleukia (ATA) and inflammatory bowel disease [13]. Similarly to other TCHA, T-2 toxin inhibits protein synthesis [14] by lowering of DNA and RNA synthesis. T-2 toxin binding to peptidyl transferase enzyme generates disorders in the translation [15]. Like other TCA, T-2 toxin activates mitogen-activated protein kinase (MAPKs) by a mechanism called the “ribotoxic stress response.” This mechanism drives both cytokine gene expression and apoptosis in macrophages [16]. An important nonribosomal effect of T-2 toxin is the intensive fast free radical production. The oxidative stress induces mitochondrial DNA damage, high lipid peroxidation, and disorders in inflammatory pathways and cell signaling [17]. Impact on protein synthesis causes leukopenia, depletes cells in lymphoid organs, and inhibits erythropoiesis in bone marrow and the spleen [18,19]. The immune system is particularly sensitive to T-2 toxin due to its damaging effects on the thymus and spleen [20]. T-2 toxin has cytotoxic effects [21]; it significantly impairs the production of antibodies [22], decreases the lymphocyte proliferative response [23], and disrupts the maturation of dendritic cells [24].

Pigs are considered as the most susceptible species to TCH contamination [25]. Pigs are a particularly interesting object of research on mycotoxins because the porcine diet is rich in cereals which are natural mycotoxin sources. In addition, porcine and human immune systems share many anatomical and physiological similarities [26], and the results of research conducted on pigs can be extrapolated to human subjects. Studies of young animals provide valuable information about mycotoxin effects on the development of immune mechanisms in gut-associated lymphoid tissue (GALT) function.

GALT, the mucosal immune system that regulates the local immune response, is the largest set of immune cells in the body and comprises organized lymphoid tissues to which we can include the Peyer’s patches (PP), isolated lymphoid follicles, and mesenteric lymph nodes (MLN) [27]. PP are covered M cells which sample antigen directly from the lumen and deliver it to antigen-presenting cells [28]. Dendritic cells and macrophages can also directly sample the lumen by extending dendrites through transcellular M cell-specific pores. T cells, B cells, and memory cells are stimulated upon encountering antigens in PP. These cells then pass to the MLN where the immune response is amplified. Activated lymphocytes pass into the blood stream via the thoracic duct and travel to the gut where they carry out their final effector functions (lamina propria). The maturation of B lymphocytes takes place in the PP. The lamina propria (LP) with macrophages, mast cells, dendritic cells, neutrophils, and lymphocytes is generally considered an effector site in the GALT [29]. After immune induction, the LP performs the function as the regulator of immune responses in the intestine [30] and the LP lymphocytes, in addition to effector function, also have a role in immunoregulation [31]. Intraepithelial lymphocytes (IEL) play a similar role. IEL are a heterogeneous subclass of T cells, integrated in the epithelial layer [32] and functionally having immunoregulatory and cytolytic functions.

Most mycotoxins enter the body via the gastrointestinal tract, and they can modulate the mucosal intestinal immune system. Studies indicate that fumonisin FB1 decreases IL-8 expression [33] and thus causes a reduction in the recruitment of inflammatory cells in the intestine in response to an infection [34]. Macrophages, B and T lymphocytes, and NK cells are very sensitive to deoxynivalenol (DON) and growing evidence indicates that the DON can have a marked impact on cytokine secretion, increase cell apoptosis, and suppress the antibody response [35]. Disturbances in synthesis and secretion of cytokines by immune cells located in the Peyer’s patches after exposure to DON are the cause of an increase in IgA production and a decrease in IgG and IgM [35]. In pigs, the weaning period and the accompanying changes in rearing conditions, in particular, the transition from liquid to solid feed, induce considerable changes in GALT function. Weaning and successive stages of growth are characterized by changes in T cells, B cells, monocytes/macrophages, and antigen-presenting cells (APC cells). Cytokines play a critical role in the regulation of immune and inflammatory responses, and they are produced not only by lymphocytes, dendritic cells, and macrophages, but also by cells which, in the classical approach, are not components of the immune system, such as intraepithelial cells (IEC) [36].

Lymphocyte subpopulations differ in their susceptibility to T-2 toxin. Double-positive CD4+CD8+ T cells [37] are B lymphocyte precursors [38] and highly sensitive to T-2 toxin. Intestinal epithelial cells play an important role in the local immune response of the gastrointestinal tract. In vitro studies into IEC-6, Caco-2, and HT-28 cell lines demonstrated that intestinal epithelial cells secrete chemokines which promote communication between the epithelium, leukocytes, and the adjacent mucosal cells. According to research, T-2 toxin enhances the expression of IL-12 and TNF-α and decreases the expression of IL-1β in peritoneal macrophages and T cells in lymph nodes [39]. Previous observations also revealed that T-2 toxin increases IL-2 expression and decreases the expression of IFN-γ in pigs.

Exposure to low doses of many mycotoxins can potentially stimulate or suppress immune functions such as lymphocyte proliferation or cellular and humoral immunity, subject to the administered dose and period of exposure [40]. The no-observed-adverse-effect level (NOAEL) and lowest-observed-adverse-effect level (LOAEL) values for most mycotoxins have been identified. In pigs, the LOAEL of T-2 toxin was determined at 29 µg/kg BW, whereas the NOAEL value could not be identified [11].

The aim of this study was to determine the effect of T-2 toxin administered at 50% of the LOAEL (approx. 14.5 µg/kg BW) on: (i) the percentage of T cells with surface markers CD2+ (T cells), CD4+ (Th helper cells), CD8+ (cytotoxic T cells), CD4+CD8+ (double-positive T cells), and CD5-TCR+ (TCRγδ+ cells); (ii) the percentage of B cells with surface markers CD5+CD8- (B1 cells) and CD21+ (B2 cells); (iii) the secretion of proinflammatory (IFN-γ, IL-1β, IL-2, IL-12/23p40), anti-inflammatory, and regulatory (IL-4, IL-10, TGFβ, IL-17A) cytokines in the porcine ileal wall.

## 2. Results

### 2.1. The Effect of T-2 Toxin on the Percentage of Lymphocyte Subpopulations

#### 2.1.1. T Lymphocyte Populations

**Figure 1:**
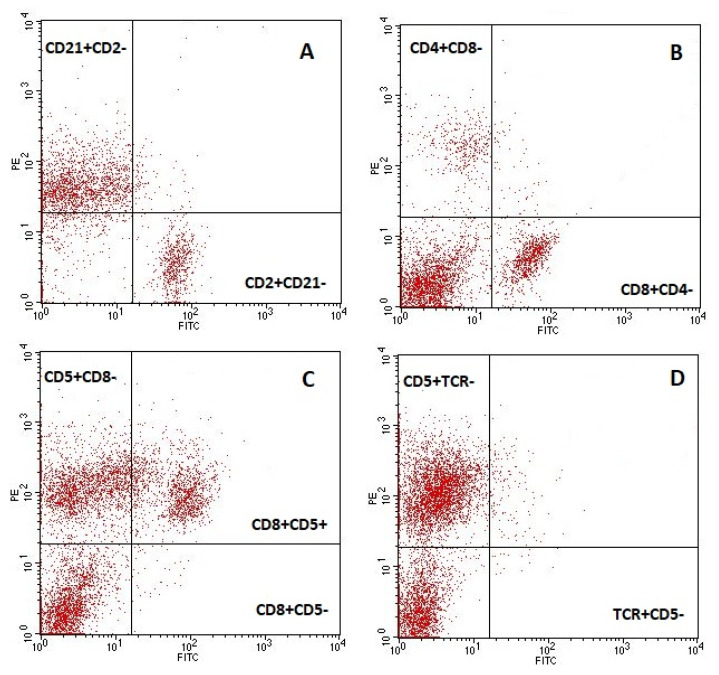
Representing dot plots of lymphocyte subpopulations: (**A**) included lymphocytes T- CD2+ and lymphocytes B- CD21+; (**B**) lymphocytes Th- CD4+CD8- and Tc- CD8+CD4- are shown; (**C**) CD8+CD5-are NK cells; (**D**) CD5+TCR+ are TCRαβ lymphocytes and CD5- TCR+ are TCRγδ lymphocytes.

The flow cytometric analysis was performed using a FACSCalibur flow cytometer and the Cell QuestTM program (BectonDickinson, Franklin Lakes, NJ, USA). A total of 10,000 cells of each sample were acquired. Lymphocytes were gated based on the FSC-area (FSC-H) vs. SSC-high (SSC-H) dot plots, and lymphocyte subpopulations were identified based on the fluorescence intensity of dot plot quadrant statistics. The representing dot plots depicting lymphocyte subpopulations are shown in Figure 1.

**Figure 2:**
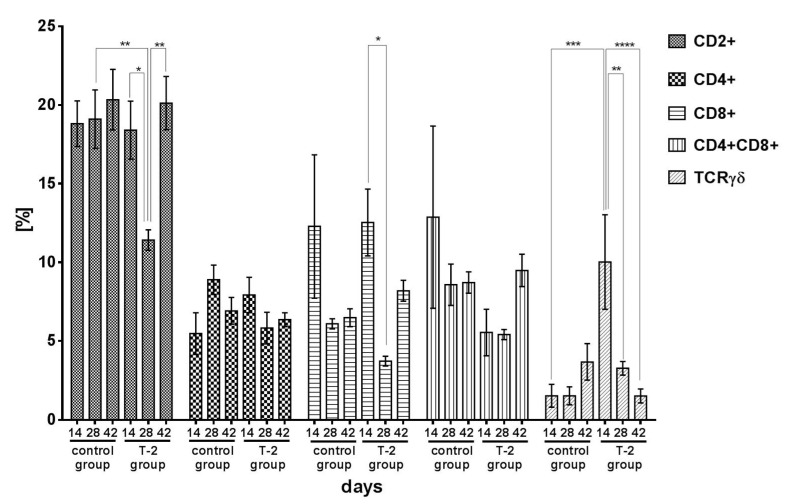
Cytometric analysis of CD2+, CD4+, CD8+, CD4+CD8+, TCRγδ+ cells isolated from porcine ileal wall in the control group and group T-2 on days 14, 28, and 42 (pg/mg protein; *n* = 5 each). The results are presented as means ± SEM. Statistically significant differences were determined at * *p* < 0.05, ** *p* < 0.01, *** *p* < 0.001, and **** *p* < 0.0001.

The average percentage of the CD2+ T cells subpopulation in the control increased from 18.90% (day 14) to 20.60% (day 42) during the six-week experiment. In group T-2, a significant decrease in the percentage of CD2+ T cells was observed during the first 28 days of exposure to T-2 toxin. On day 28, the percentage of CD2+ T cells was significantly (*p* < 0.01) higher in the control group than in group T-2. In group T-2, the percentage of CD2+ T cells was lowest on day 28 (11.40%), and it differed significantly from the values noted on days 14 and 42 (*p* < 0.05 and *p* < 0.01, respectively) (Figure 2).

The percentage of CD4+ Th helper cells did not change significantly in either group and ranged from 5.60% (day 14) to 8.98% (day 28) in the control group, and from 7.93% (day 14) to 5.82% (day 28) in group T-2 (Figure 2).

The percentage of CD8+ cytotoxic T cells (Tc) tended to decrease in the control group, but the noted differences were not statistically significant. In group T-2, the percentage of CD8+ Tc decreased significantly (*p* < 0.05) on day 28 relative to day 14. The percentage of CD8+ Tc increased on day 42 (Figure 2).

The percentage of double-positive CD4+CD8+ T cells in the ileal wall tended to decrease in the control group. In group T-2, the percentage of double-positive CD4+CD8+ T cells clearly decreased on days 14 and 28, and it increased rapidly on day 42, but the noted differences were not statistically significant (Figure 2).

In the control group, the percentage of TCRγδ+ lymphocytes remained low throughout the experiment, and it increased from 1.60% on day 14 to 3.65% on day 42. In group T-2, the percentage of TCRγδ+ cells was high in the first 14 days of exposure (10.00%), and it decreased significantly to 3.30% on day 28 (*p* < 0.01) and to 1.50% on day 42 (*p* < 0.001) (Figure 2).

#### 2.1.2. B Lymphocyte Populations

**Figure 3:**
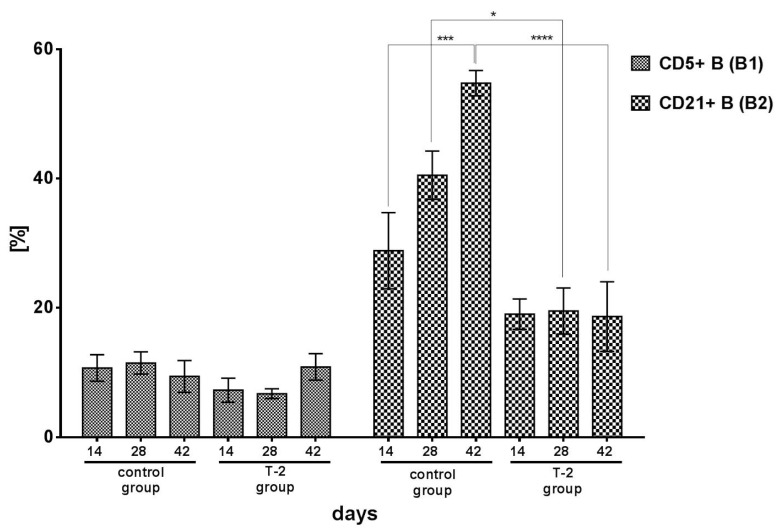
Cytometric analysis of CD5+ B (B1) cells and B CD21+ (B2) cells isolated from porcine ileal wall in the control group and group T-2 on days 14, 28, and 42 (pg/mg protein; *n* = 5 each). The results are presented as means ± SEM. Statistically significant differences were determined at * *p* < 0.05, *** *p* < 0.001, and **** *p* < 0.0001.

The percentage of CD5+ B (B1) cells did not differ significantly between groups or experimental days. Despite the above, the lowest percentage of CD5+ cells was noted in group T-2 on day 14 (7.28%) and day 28 (6.74%) (Figure 3).

In the control group, a significant (*p* < 0.001) increase in the percentage of CD21+ B (B2) cells was noted during the experiment, from 29.40% on day 14 to 55.20% on day 42. In group T-2, the percentage of B2 lymphocytes remained low and stable throughout the experiment at 19.00% on day 14, 19.94% on day 28, and 18.63% on day 42. The exposure to T-2 toxin significantly suppressed the percentage of CD21+ B2 cells. On days 28 and 42, the percentage of B2 lymphocytes in group T-2 was significantly lower than in the control group (*p* < 0.001 and *p* < 0.0001, respectively) (Figure 3).

### 2.2. The Effect of T-2 Toxin on Cytokine Secretion

#### Proinflammatory Cytokines

**Figure 4:**
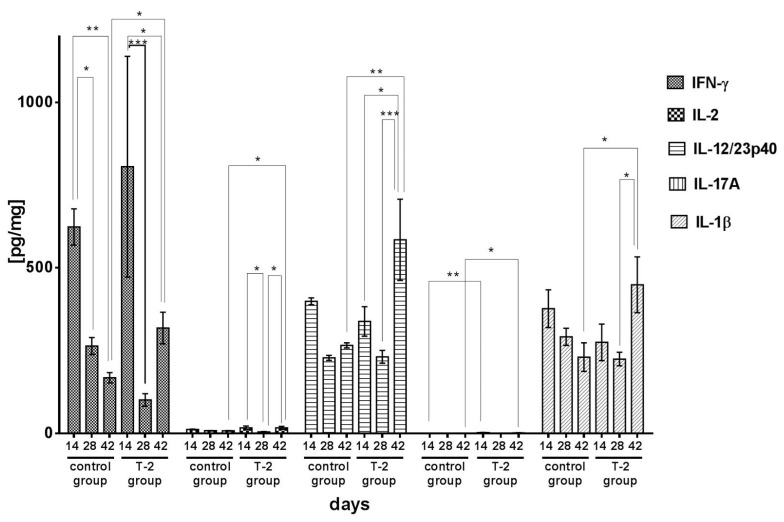
Proinflammatory cytokines concentrations in the porcine ileal wall in the control group and group T-2 on days 14, 28, and 42 (pg/mg protein; n = 5 each). The results are presented as means ± SEM. Statistically significant differences were determined at * *p* < 0.05, ** *p* < 0.01, and *** *p* < 0.001.

The concentration of IFN-γ in the ileal wall of control group and group T-2 animals tended to decrease throughout the experiment. The mean concentration of IFN-γ was determined at 623.50 pg/mg on day 14, and it was significantly higher than on days 28 and 42 (*p* < 0.05 and *p* < 0.01, respectively). IFN-γ levels peaked (805.90 pg/mg) in group T-2 after 14 days of exposure. On days 28 and 42, the analyzed parameter decreased significantly to 101.60 pg/mg (*p* < 0.001) and 318.20 pg/mg (*p* < 0.05), respectively (Figure 4).

In the control group, the concentration of IL-1β was characterized by a linear decrease from 377.50 pg/mg on day 14 to 231.60 pg/mg on day 42. In contrast, in group T-2, the concentration of IL-1β increased from 275.10 pg/mg on day 14 to 448.60 pg/mg on day 42. On day 42, the analyzed parameter was significantly (*p* < 0.05) higher in group T-2 than in the control group (Figure 4).

The concentration of IL-2 in the ileal wall of control group animals continued to decrease throughout the experiment, from 12.70 pg/mg on day 14 to 8.30 pg/mg on day 42, but the differences between mean values were not significant. In group T-2, the concentration of IL-2 was significantly (*p* < 0.05) higher on day 14 than on day 28. The analyzed parameter increased significantly (*p* < 0.05) on day 42 relative to day 28 (Figure 4).

In the control group, the concentration of IL-12/23p40 in the ileal wall ranged from 229.50 pg/mg (day 28) to 397.85 pg/mg (day 14). In group T-2, the concentration of IL-12/23p40 was lowest on day 28 (231.50 pg/mg), and a significant (*p* < 0.001) increase was noted on day 42 (584.80 pg/mg). The above increase was also significant (*p* < 0.01) relative to the value noted in the control group on the last day of the experiment (Figure 4).

In the control group, the mean concentration of IL-17A in the ileal wall ranged from 0.83 pg/mg (day 14) to 1.05 pg/mg (day 28), and it was considerably lower than in group T-2 during the entire experiment. The concentration of IL-17A peaked in group T-2 on day 14 (2.37 pg/mg). In animals exposed to T-2 toxin for 14 and 42 days, IL-17A levels were significantly higher (*p* < 0.01 and *p* < 0.05, respectively) than in the control group (Figure 4).

### 2.3. Anti-Inflammatory and Regulatory Cytokines

In the control group, a minor decrease in the concentration of IL-4 was noted during the experiment, from 72.40 pg/mg on day 14 to 56.30 pg/mg on day 42, but no significant differences were found between mean values. IL-4 levels also decreased in group T-2. On day 14, the mean concentration of IL-4 in the ileal wall of the animals exposed to T-2 toxin was significantly (*p* < 0.001) higher than in the control group. In group T-2, the concentration of IL-4 decreased significantly (*p* < 0.0001) on day 28 relative to day 14. On day 42, the analyzed parameter was significantly (*p* < 0.01) lower than on day 14, but significantly (*p* < 0.05) higher than on day 28 (Figure 4).

**Figure 5:**
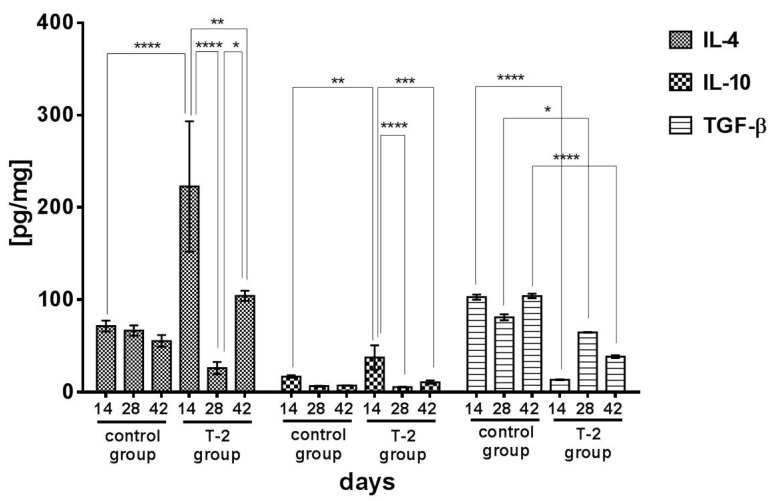
Anti-inflammatory and regulatory cytokines concentrations in the porcine ileal wall in the control group and group T-2 on days 14, 28, and 42 (pg/mg protein; *n* = 5 each). The results are presented as means ± SEM. Statistically significant differences were determined at * *p* < 0.05, ** *p* < 0.01, *** *p* < 0.001, and **** *p* < 0.0001.

In the control group, the mean concentration of IL-10 in the ileal wall decreased from 17.00 pg/mg on day 14 to 6.50 pg/mg on day 28, and a minor increase to 7.20 pg/mg was observed on day 42. A similar change trend was noted in group T-2, but on day 14, the mean concentration of IL-10 (37.50 pg/mg) was significantly (*p* < 0.01) higher than in the control group. In group T-2, a minor increase in IL-10 levels was observed between days 28 and 42, but the studied parameter was significantly lower on days 28 and 42 than on day 14 (*p* < 0.0001 and *p* < 0.001, respectively) (Figure 5).

In the control group, the mean concentration of TGFβ in the ileal wall ranged from 80.95 pg/mg on day 28 to 104.00 pg/mg on day 42, and it was considerably higher than in the experimental group. After 28 days of exposure to T-2 toxin, TGFβ levels in the experimental group reached 64.69 pg/mg. The concentration of TGFβ was lower in group T-2 than in the control group on all analyzed days of the experiment (*p* < 0.0001, *p* < 0.05, *p* < 0.0001, respectively) (Figure 5).

## 3. Discussion

A strong immune system is essential for protecting living organisms against pathogens, but the same mechanisms activated in response to dietary proteins or commensal bacteria can lead to the development of chronic diseases. A complex interplay of regulatory mechanisms in the intestinal immune system generally prevents such interactions [27]. Immune responses are initiated in sites such as Peyer’s patches with T and B lymphocytes, dendritic cells, and macrophages that secrete cytokines, creating a unique cellular and cytokine environment. Intestinal epithelial cells also participate in the activation of immune system cells. Two distinct lymphocyte populations are formed in the intestines: intraepithelial lymphocytes (IEL), accounting for 10–30% of the lymphocyte population, which suppress immune responses to antigens and exert cytotoxic effects on virus-infected cells (Tc), and lamina propria lymphocytes (LPL), mostly B cells, plasma cells producing mainly dimeric IgA, and memory cells, accounting for 70–90% of the lymphocyte population.

There is a general scarcity of published research into the effects of low oral doses of T-2 toxin on the intestinal immune system. Therefore, this study evaluated the influence of T-2 toxin administered at 50% of the LOAEL (approximately 14.5 µg/kg BW) [9] on the percentage of T cells with surface markers CD2+ (total percentage of T cells), CD4+ (Th helper cells), CD8+ (cytotoxic T cells), CD4+CD8+ (double-positive T cells), and CD5-TCR+ (TCRγδ+ lymphocytes, intraepithelial lymphocytes), the percentage of B cells with surface markers CD5+CD8- (B1 cells) and CD21+ (B2 cells), and the secretion of proinflammatory (IFN-γ, IL-1β, IL-2, IL-12/23p40), anti-inflammatory, and regulatory cytokines (IL-4, IL-10, TGFβ, IL-17A) in the porcine ileal wall.

The present study was conducted on prepubertal pigs because this animal species is highly sensitive to TCH and is regarded as a model species for biomedical research due to physiological and immunological similarities to humans [41]. Moreover, the anatomy, nutritional requirements, and hypersensitive responses of young pigs are also similar to those observed in children [42], which is yet another important consideration. An attempt was made in this study to determine whether exposure to a low dose of T-2 toxin over different periods of time affects the development of the intestinal immune system, the recognition of potentially beneficial and harmful antigens, and the activation of tolerance to food antigens in prepubertal gilts.

Exposure to T-2 toxin increased the secretion of cytokines, in particular, proinflammatory cytokines, in the ileal wall. The similar effect causes a low level of the deoxynivalenol (DON) member of the TCH type B, which induces mRNAs for TNF-α and IL-6 in macrophages [43]. DON also induces secretion of IL-2, IL-4, IL-6, IL-8, and TNFα by lymphocytes [44]. In our study, the concentration of IFN-γ increased over time and peaked on day 42 (Figure 4). The in vivo effect of FB1 on the production of IFN-γ has also been reported by Taranu et al. [45]. They found increased expression of IFN-γ in the mesenteric lymph node and spleen of pigs. An increase was also noted in the levels of IL-1β, IL-2, IL-12/23p40, and IL-17A. Interestingly, a growing trend was also observed in the concentrations of anti-inflammatory cytokines, including IL-10 and IL-4. What is important is that IL-10 is produced by both Tregs and rTh17 to regulate inflammation [46]. The proinflammatory effects of T-2 toxin were confirmed by a decrease in the secretion of TGFβ (Figure 5), whose concentration continued to decrease on successive days of the experiment relative to the control group. It should be noted that TGFβ has immunosuppressive properties, and low levels of this cytokine induce the Th17 cell response and the secretion of IL-17A [47]. Other authors also reported correlations between the dose of T-2 toxin and other TCH and the concentrations of secreted cytokines. According to Schuhmacher-Wolz et al. [48], unlike high doses of TCH (mg/kg BW), low doses (µg/kg BW) generally stimulate the immune system. Similar effects were noted by Ahmadi and Riazipour [39] in in vitro cultures of mouse peritoneal macrophages and lymph node cells exposed to T-2 toxin doses of 0.001 µg/mL to 100 µg/mL. DON in low concentrations induces expression of early response and the mRNA proinflammatory genes levels [49]. The concentrations of IL-1β, IL-2, IL-4, IL-10, IL-12, TNFα, and IFN-γ increased in response to T-2 toxin doses lower than 0.1 µg/mL, but not under exposure to doses higher than 1 µg/mL [28]. Li et al. also observed an increase in the secretion of proinflammatory cytokines in an avian macrophage cell line in response to T-2 toxin doses of 1–5 ng/mL [50].

In addition to immunocompetent cells, enterocytes also play a very important role in the induction of tolerance to food antigens in the intestinal lumen [51]. Intestinal epithelial cells secrete cytokines which suppress the immune system and increase tolerance to food antigens, including thymic stromal lymphopoietin (TSLP), TGFβ, and retinol metabolites (retinoids) that induce the development of dendritic cells [52]. Dendritic cells trigger the activation of antigen-specific regulatory T cells (Tregs) [53,54,55] and promote immunoglobulin class switching to IgA in B2 lymphocytes regardless of CD4+ Th cells. TGFβ plays a key role in the induction of IgA synthesis by B2 cells, and the induction of CD4+ regulatory (iTreg) T cells with the FoxP3 (CD4+CD25+FoxP3+) transcription factor from CD4+CD25- FoxP3 naive T cells [56].

In the group of animals exposed to T-2 toxin, the decrease in TGFβ secretion could be one of the factors that triggered the production of proinflammatory cytokines and impair natural tolerance to food antigens in the intestine. Dendritic cells, which exhibit tolerogenic properties, migrate to mesenteric lymph nodes and induce the production of iTreg cells and TGFβ secretion [57]. The proinflammatory properties of T-2 toxin suppress the activity of tolerogenic dendritic cells. In addition to macrophages, these cells are the main source of IL-12/23 p40 and IL-1β. In this study, dendritic cells were secreted in large amounts in the ileal wall of gilts exposed to T-2 toxin, and their concentrations continued to increase during the experiment. The above cytokines, similarly to IL-17A, regulate the body’s response to infections, including to lipopolysaccharides (LPS) in Gram-negative bacteria. Similar activity is demonstrated by B1 lymphocytes (CD5+CD8-) (Figure 3) which produce immunoglobulin M. One of the negative effects exerted by a low dose of T-2 toxin on GALT was a decrease in the percentage of B2 lymphocytes (CD21+), which could decrease the synthesis of IgA, compromise the integrity of the intestinal epithelial barrier, and impair tolerance to food antigens.

The results of the present study indicate that T-2 toxin administered at 50% of the LOAEL exerts stimulatory and proinflammatory effects on the immune system. Similar observations were made by Ahmadi and Riazipour [39], who found that low doses of T-2 toxin increase the synthesis of interleukin-12 and TNF-α in mice peritoneal macrophages. However, our study investigated young animals whose tolerance to food antigens should increase during the development of immunity. Unfortunately, this process was compromised in the group of animals exposed to T-2 toxin. The above led to inflammation which impaired nutrient absorption. Similar responses have been observed under exposure to other trichothecene mycotoxins. Satrartoxin G, roridin A, verrucarin A, and T-2 toxin can activate caspase-3 as well as caspase-1, whose activation is mediated by the inflammasome protein complex (e.g., in macrophages). The above can increase the secretion of IL-1β and IL-18 [58]. Trichothecenes, including T-2 toxin, interact with nucleic acids at the cellular level. They trigger the ribotoxic stress response, which leads to depurination and cleavage of 28S and 18S rRNA [59], and activation of mitogen-activated protein kinase (MAPK), protein kinase R (PKR), and JNK/p38 kinase [60]. These kinases increase the expression of genes encoding proinflammatory cytokines IL-1β, IL-6, and TNFα, as well as chemokines IL-8 and the macrophage inflammatory protein-2 (MIP-2) in monocytes/macrophages [61]. The antigens present in the intestinal lumen and those reaching the intestine indirectly via the blood stream were effectively absorbed in the jejunum under exposure to deoxynivalenol (DON) [62]. A reduction in the number of goblet cells in the intestines and a decrease in the expression of tight junction proteins (claudin-3, claudin-4, zonula occludens-1 (ZO-1) protein, and occludin) were also noted [33]. The expression of tight junction proteins is related to the activity of MAPK. The above correlations were found mostly in studies of DON, which is less toxic than T-2 toxin [63]. In the current study, similar conclusions can be formulated based on the increase in the concentration of IFNγ (Figure 4), which also stimulates extracellular signal-regulated kinases (ERK) and c-Jun N-terminal kinases (JNK) [64]. The activation of MAPK also decreases the expression of tight junction proteins claudin-3 and claudin-4 [33].

In the studied pigs, the immune proinflammatory response of the ileal wall was similar to that noted in humans with inflammatory bowel disease (IBD). Mycotoxins are environmental factors that are often associated with Crohn’s disease and ulcerative colitis [65]. The concentrations of proinflammatory cytokines also increase in other autoimmune disorders, including celiac disease. In this study, IL-17A and IFNγ continued to increase on successive days of exposure to T-2 toxin (relative to day 28), and they were secreted by immunocompetent cells at a similar rate. Inversely proportional changes in the concentrations of TGFβ and IFNγ (decrease in TGFβ secretion and increase in IFNγ production) corroborate the above observations. An increase in the percentage of CD8+ cytotoxic T cells which may secrete IFNγ as well as IL-17A [55] also leads to various types of hypersensitivity in autoimmune disorders [66]. Crohn’s disease and celiac disease are type IV hypersensitivity reactions which involve IFNγ and cytotoxic lymphocytes. Similar effects are observed in contact hypersensitivity induced by haptens, which are nonimmunogenic, low-molecular-weight chemicals (below 1 kDa). Haptens can interact with the immune system when they are covalently attached to carrier molecules, including other proteins [67].

The proinflammatory properties of T-2 toxin can also be attributed to its ability to attach protein sulfhydryl groups, including enzymes of the oxidoreductase family. Trichothecenes can affect mitochondrial metabolism, in particular, enzymes that participate in the tricarboxylic acid cycle and the oxidative phosphorylation (OXPHOS) process. T-2 toxin inhibits succinate dehydrogenase, which catalyzes the conversion of succinic acid to fumaric acid [68]. This process leads to the accumulation of succinic acid, an inflammatory marker [69,70] that influences the expression of hypoxia-inducible factor-1α (HIF1α). Macrophages that are classically activated by LPS (M1 macrophages) as well as Th1 and Th17 lymphocytes undergo metabolic reprogramming during which the Krebs cycle is disrupted by deficiencies in succinate dehydrogenase and isocitrate dehydrogenase, which leads to the accumulation of succinate and isocitrate. The described organic acids in the structure of tricarboxylic acid stabilize HIF-1α. Macrophages secrete IL-1β and alter the phenotype characterized by high activity of the glycolytic pathway and the pentose phosphate pathway. In Th1 and Th17 lymphocytes, metabolic reprogramming towards glycolysis is also associated with the secretion of IFN-γ and IL-17A. The presence of proinflammatory cytokines in the ileal wall is also observed in IBD, including Crohn’s disease. T-2 toxin inhibits mitochondrial metabolism, disrupts the electron transport chain, and promotes the release of reactive oxygen species that increase oxidative stress and further contribute to intestinal inflammation.

## 4. Conclusions

In prepubertal gilts orally administered T-2 toxin at 50% of the LOAEL, immune homeostasis in the ileal wall was compromised between day 14 and day 42 of exposure. The observed increase in the concentrations of proinflammatory cytokines IL-1β, IL-2, IL-12/23p40, IL-17A, and IFNγ and regulatory cytokines IL-4 and IL-10, and the decrease in the secretion of anti-inflammatory cytokine TGF-β indicate that T-2 toxin disrupted the development of tolerance to food antigens. When administered at a dose of approximately 14.5 µg/kg BW, T-2 toxin stimulated the cellular response by increasing the percentage of CD8+ cytotoxic T cells and decreasing the concentration of CD21+ B2 cells. An increase in IL-1β and IL-12/23p40 levels in the ileal wall of gilts and ongoing inflammation suggest that T-2 toxin compromises the integrity of the intestinal mucosal barrier. The decrease in the percentage of Tγδ intraepithelial lymphocytes indirectly corroborates the above observation.

## 5. Materials and Methods

### 5.1. Animals and Procedures

All experimentation procedures involving animals were consistent with the Polish law and had been approved by the Local Bioethics Committee on Animal Research in Olsztyn, Poland, under decision No. 14/2008 of 22 January 2008.

The experiment was conducted on 30 Polish Large White prepubertal gilts aged eight weeks, with a body weight of 18–20 kg (Research project No. N N308 237936 of 24 April 2009, financed by the Polish National Science Centre). The animals were randomly divided into two groups and were kept in separate pens with ad libitum access to water. The adaptation period lasted seven days. Gilts were fed a complete commercial cereal-based diet whose composition is presented in Table 1. To account for the changes in nutrient requirements during growth, the animals were fed Diet 1 until 25 kg BW, followed by Diet 2 until the end of the experiment. The experimental group (*n* = 15) was administered T-2 toxin at a daily dose of 14.5 µg/kg BW (T-2 Toxin, T4887, Sigma-Aldrich, Poznań, Poland). The daily dose of T-2 toxin was modified to account for the increase in the body weight of animals. The toxin was administered orally in gel capsules, once a day, before morning feeding. At the same time, control group gilts (*n* = 15) were administered empty gel capsules to maintain identical levels of handling stress in all animals.

To rule out the presence of mycotoxins in the administered diets, every feed batch was analyzed for the presence of T-2 toxin, aflatoxin B1, ochratoxin A, zearalenone, alpha-zearalenone, and deoxynivalenol with the use of high-performance liquid chromatography (HPLC) with FLD and/or UV detection (Agilent Technologies 1100, Santa Clara, CA, USA). None of the above mycotoxins were identified above the limit of detection in the administered feeds.

The experiment lasted 42 days. Cytokine secretion and the percentage of lymphocyte subpopulations were determined on experimental days 14, 28, and 42. Every two weeks, five gilts selected randomly from each group were administered azaperone by IM injection at 4 mg/kg BW (Stresnil, Beerse, Belgium) and were euthanized with an IV-administered lethal dose of sodium pentobarbital (0.6 mL/kg BW) (Morbital, Biowet Puławy, Poland) after 15 min.

### 5.2. Tissue Sampling and Specimen Preparation

Immediately after euthanasia, 5 cm long segments of the ileal wall were sampled approximately 2 cm before the ileocecal valve. Tissue samples for the cytokine analysis were stored at a temperature of −80 °C.

Ileal samples for the cytometric analysis of selected lymphocyte populations (Table 2) were rinsed in phosphate-buffered saline (PBS, pH 7.4, 0.1 M) and finely chopped to remove lymphatic cells with PBS. The cells were centrifuged to remove excess PBS, the pellet was distributed into test tubes, and the cells were counted in the Fuchs-Rosenthal counting chamber. Cell suspensions containing 10^6^ cells/μL were prepared for immunophenotyping. Lymphocyte subpopulations were labeled by dual staining with a combination of primary antibodies (Table 3). The antibodies labeled with fluorescent dyes fluorescein (FITC) and phycoerythrin (PE) were the secondary antibodies (Table 4). Three controls were used in immunophenotyping: without fluorochromes, and with PE and FITC fluorochromes, but without primary antibodies. Stained cells were fixed in 1% formalin to preserve the samples for 24 h. The prepared specimens were immediately transported to the Flow Cytometry Laboratory of the Institute of Laboratory Diagnostics at the Department of Pathology and Veterinary Diagnostics, Faculty of Veterinary Medicine of the Warsaw University of Life Sciences. In the laboratory, the specimens were analyzed in the FACScalibur flow cytometer, and data were processed in the Cell QuestTM program (Becton Dickinson, San Jose, CA, USA).

### 5.3. Determination of Protein Levels and Cytokine Concentrations

Ileal samples of 1 g each were homogenized in an extraction buffer containing PBS (Sigma-Aldrich, Saint Louis, MO, USA), 0.5% sodium citrate (POCH, Poland), 0.05% Tween 20 (Sigma Aldrich, Saint Louis, MO, USA) and protease inhibitors (Ref 11 697 498 001, Roche, Poland) with the use of Omni TipsTM plastic disposable homogenizer probes (Omni International). The homogenate was centrifuged (8600× *g*) for 1 h in the Eppendorf 5804R centrifuge, and the supernatant was distributed into test tubes and stored at a temperature of −80 °C until analysis.

The concentration of protein in the extract was determined according to the Bradford method with minor modifications [71], and the results were used as a reference in the cytokine analysis. Cytokine concentrations were expressed in pg/mg protein.

The content of IFN-γ, IL-1β, IL-2, IL-12/23p40, IL-4, IL-10, TGFβ, and IL-17A was determined with the use of commercial ELISA kits (Table 5) in a microplate spectrophotometer (TECAN Infinite M200, Männedorf, Switzerland).

### 5.4. Statistical Analysis

The results were processed in Excel (Microsoft, Redmond, WA, USA) and Graph Pad Prism 5 (GraphPad Software, San Diego, CA, USA) programs. Mean values and standard error of the mean (SEM) were calculated for all studied groups. The distribution of the population was determined with the Kolmogorov–Smirnov test. The results were analyzed statistically using an unpaired *t*-test and one-way ANOVA at a significance level of *p* < 0.05.

## Figures and Tables

**Table 1:** Composition of diets fed to gilts (per 1 kg of feed).

Ingredients	Unit	Diet 1	Diet 2
Metabolizable energy	MJ	13.80	13.00
Total protein	%	6.00	16.00
Crude fiber	max%	2.63	4.94
Sodium	%	0.21	0,18
Calcium	min%	0.73	0.58
Total phosphorus	min%	0.65	0.73
Lysine	%	1.27	1.04
Methionine + cystine	%	0.83	0.64
Threonine	%	0.72	0.59
Tryptophan	%	0.26	0.20
Vitamin A	IU	15,000	13,500
Vitamin D_3_	IU	2000	2000
Vitamin E	IU	100	60
Phytase	present	+	+
Enzymes	present	+	+
Flavor	present	+	-
Acidifier	present	+	+

**Table 2:** Analyzed lymphocyte subpopulations.

Antigen	Subpopulation
CD2+	T cells
CD4+	Th cells
CD8+	Cytotoxic T cells
CD4+CD8+	Double-positive T cells
CD5-TCR+	TCRγδ+ cells, intraepithelial lymphocytes
CD5+CD8-	B1 cells
CD21+	B2 cells

All reagents for immunophenotyping were supplied by Pharmingen (San Diego, CA, USA).

**Table 3:** Primary antibodies used in flow cytometry.

Symbol	Detected Antigen	Immunoglobulin Class	Catalog Number
P1	CD2	IgG2a	MSA4
P2	CD4	IgG2b	74-12-4
P3	CD5	IgG1	PG114A
P4	CD8α	IgG2a	76-2-11
P5	CD21	IgG1	BB6-11C9
P6	TCR1-N7	IgG1	86D

All reagents for immunophenotyping were supplied by Pharmingen (San Diego, CA, USA).

**Table 4:** Secondary antibodies used in flow cytometry.

Symbol	Immunoglobulin or Ligand Class	Dye	Catalog Number
S1	Mouse IgG1	Phycoerythrin (PE)	550083
S2	Mouse IgG2a	Fluorescein isothiocyanate (FITC)	553390
S3	Mouse IgG2b	Biotin	550333
S-PE	Biotin	PE	554061

All reagents for immunophenotyping were supplied by Pharmingen (San Diego, CA, USA).

**Table 5:** ELISA kits for determining cytokine concentrations in the porcine ileum.

Antigen	ELISA Kit and Catalog Number	Manufacturer, Country	Assay Range pg/mL
IFN-γ	Porcine IFN-gamma DuoSet ELISA, DY985	R&D Systems Inc., Minneapolis, MN, USA	62.5–4.000 Intra-assay CV < 3.4% Inter-assay CV < 4.6%
IL-1β	Porcine IL-1 beta/IL-1F2 DuoSet ELISA, DY681	R&D Systems Inc., Minneapolis, MN, USA	62.5–4.000 Intra-assay CV < 1.1% Inter-assay CV < 3.2%
IL-2	Swine IL-2 CytoSet™ CSC124	Invitrogens™, Camarillo, CA, USA	35.6–570 Intra-assay CV < 4.48% Inter-assay CV < 5.02%
IL-12/23p40	Porcine IL-12/IL-23 p40 DuoSet ELISA, DY912	R&D Systems Inc., Minneapolis, MN, USA	78.1–5.000 Intra-assay CV < 3.67% Inter-assay CV < 4.25%
IL-4	Porcine IL-4 DuoSet ELISA, DY654	R&D Systems Inc., Minneapolis, MN, USA	156.0–10.000 Intra-assay CV < 5% Inter-assay CV < 6.69%
IL-10	Porcine IL-10 DuoSet ELISA, DY693B	R&D Systems Inc., Minneapolis, MN, USA	23.4–1.500 Intra-assay CV <3% Inter-assay CV <4.64%
TGFβ	TGF beta-1 Multispecies Matched Antibody Pair, CHC1683	Thermo Fisher Scientific, Waltham, MA, USA	62.5–4.000 Intra-assay CV < 2.9% Inter-assay CV < 5%
IL-17A	Porcine IL-17 (IL-17A) ELISA Kit, ESIL17A	Thermo Fisher Scientific, Waltham, MA, USA	16.38–4.000 Intra-assay CV < 10% Inter-assay CV < 12%

## Data Availability

Not applicable.

## Abbreviations

18S RNA: 18S ribosomal RNA; 28S RNA: 28S ribosomal RNA; B1 cells: subclass of CD5+CD8- B lymphocytes; B2 cells: subclass of CD21+ B lymphocytes; Caco-2: CACO-2 Cell Line human; CD4+: CD4+ helper T cells; CD8+: cytotoxic T cells; CD4+CD8+: double-positive T cells; CD44low: T cell populations with low expression of CD44; CD45low: T cell populations with low expression of CD45; CD5-TCR+: CD5-positive T cells; DON: Deoxynivalenol; ELISA: Enzyme-linked immunosorbent assay; ERK: Extracellular signal-regulated kinase; FITC: Fluorescein isothiocyanate; FoxP3: Forkhead box P3; GALT: Gut-associated lymphoid tissue; HIF-1α: Hypoxia-inducible factor 1-alpha; HPLC-FLD: High-performance liquid chromatography with fluorescence detection; HPLC-UV: High-performance liquid chromatography with ultraviolet detection; HT-2: HT-2 toxin; HT-28: HT-28 cells; IBD: Inflammatory bowel disease; IEC-6: Intestinal Epithelioid Cell line No. 6; IEL: Intraepithelial lymphocytes; IFN-γ: Interferon gamma; IgA: Immunoglobulin A; IL-1: Interleukin 1; IL-10: Interleukin 10; IL-12/23p40—Interleukin 12/23 with 40 subunits; IL-15: Interleukin 15; IL-17A: Interleukin 17A; IL-18: Interleukin 18; IL-1β: Interleukin 1 beta; IL-2: Interleukin 2; IL-4: Interleukin 4; IL-6: Interleukin 6; IL-8: Interleukin 8; JNK: C-Jun N-terminal kinase; LOAEL: Lowest-observed-adverse-effect level; LPL: Lamina propria lymphocytes; LPS: Lipopolysaccharides; MAP: Multiple antigenic peptide; MAPK: Mitogen-activated protein kinase; MIP-2: Macrophage inflammatory protein-2; NOAEL: No-observed-adverse-effect level; OXPHOS: Oxidative phosphorylation; PBS: Phosphate-buffered saline; PE: Phycoerythrin; PKR: Protein kinase R; RNA: Ribonucleic acid: sIgA: Secretory IgA; SLA-DQ+: SLA-DQ+ cells; SWC3+: SWC3 + cells; CD4+CD8+ T cells: Double-positive T Cells; CD8+ T cells: T cells with CD8 co-receptors; T-2: T-2 toxin; TCH: Trichothecenes; TCHA: Type A trichothecenes; TCRγδ: T cell receptor formed by gamma-delta chains; TGF-α: Transforming growth factor alpha; TGFβ: Transforming growth factor beta; Th1: Type 1 helper T cells; Th17: Th17 helper cells; TNF-α: Tumor necrosis factor α; Tδγ: Gamma delta T cells; ZO-1: Zonula occludens-1.
